# Cloning of Three Cytokinin Oxidase/Dehydrogenase Genes in *Bambusa oldhamii*

**DOI:** 10.3390/cimb45030123

**Published:** 2023-02-27

**Authors:** Chun-Yen Hsieh, Lu-Sheng Hsieh

**Affiliations:** 1 Department of Pathology and Laboratory Medicine, Shin Kong Wu Ho-Su Memorial Hospital, Taipei City 11101, Taiwan; 2 Department of Food Science, College of Agriculture, Tunghai University, Taichung 40704, Taiwan

**Keywords:** *Bambusa oldhamii*, cytokinin oxidase/dehydrogenase, genomic DNA library

## Abstract

Cytokinin oxidase/dehydrogenase (CKX) catalyzes the irreversible breakdown of active cytokinins, which are a class of plant hormones that regulate cell division. According to conserved sequences of *CKX* genes from monocotyledons, PCR primers were designed to synthesize a probe for screening a bamboo genomic library. Cloned results of three genes encoding cytokinin oxidase were named as follows: *BoCKX1*, *BoCKX2*, and *BoCKX3*. In comparing the exon-intron structures among the above three genes, there are three exons and two introns in *BoCKX1* and *BoCKX3* genes, whereas *BoCKX2* contains four exons and three introns. The amino acid sequence of BoCKX2 protein shares 78% and 79% identity with BoCKX1 and BoCKX3 proteins, respectively. *BoCKX1* and *BoCKX3* genes are particularly closely related given that the amino acid and nucleotide sequence identities are more than 90%. These three BoCKX proteins carried putative signal peptide sequences typical of secretion pathway, and a GHS-motif was found at N-terminal flavin adenine dinucleotide (FAD) binding domain, suggesting that BoCKX proteins might covalently conjugate with an FAD cofactor through a predicted histidine residue.

## 1. Introduction

Cytokinin is a phytohormone of plant growth regulators, playing an important role in the control of plant division. Many developmental events, such as shoot and root branching, leaf development, delay of senescence, and chloroplast ripening are directly regulated by the physiological functions of cytokinins [1,2,3,4,5]. In plants, the homeostasis of cytokinins is balanced in many aspects [6], including rate of de novo synthesis by isopentenyl transferase enzyme [7], rate of interconversion and transport [8], and rate of cytokinin turnover, mainly by cytokinin oxidase/dehydrogenase [9].

The *CKX* gene-encoded cytokinin oxidase/dehydrogenase enzyme (CKX, EC 1.5.99.12) catalyzes the turnover of the cytokinins, isopentenyl-adenine, zeatin, and their ribosides by a unique enzymatic reaction by cleaving and oxidizing its side chain for converting isopentenyladenine to unsaturated 3-methyl-2-butenal and adenine [2,10]. CKX enzyme activity was initially identified by Pačes and coworkers in 1971 [11] and named by Whitty and Hall in 1974 [12]. After nearly three decades, *ZmCKX1* was the first *CKX* gene independently cloned by two research groups in maize *Zea mays* [13,14]. CKX proteins are usually encoded by homologous genes in plants, such as *Arabidopsis thaliana* [15,16], *Hordeum vulgare* [17,18], *Jatropha curcas* [19], *Oryza sativa* [9,20,21], *Zea mays* [22,23], and so on. Recently, 23 *BnCKX* genes were identified in *Brassica napus* [24]. Therefore, CKX enzyme activity largely governs the utilization of cytokinins in plant cells.

Elevated cytokinin levels were linked with increased cytokinin degradation by CKX enzyme in many plants, such as *Zea mays* [25], *Triticum aestivum* [26], and *Brassica napus* [24,27], suggesting that *CKX* gene expression is manipulated by endogenous cytokinin extents [5]. Overexpression of the *AtCKX3* [28] and *OsCKX2* [9] are associated with reduced flower numbers and grain numbers, respectively. Expression of the *AtCKX1* in *Nicotiana tabacum* enhances drought and heat stress tolerance, indicating that cytokinin levels may have a positive effect on plant stress responses [29,30,31]. Under salinity condition, rice yield penalty is reduced by knockdown of the *OsCKX2* gene as well as increased inflorescence meristem cytokinin extent [32].

Alternation in CKX enzymatic activity changes cytokinin concentrations in cells and tissues. CKX enzymes play key roles in contributing to the regulation of cytokinin-dependent processes and in controlling local cytokinin level [33]. Functions of CKX proteins can be regulated by several post-translational modifications, e.g., glycosylation, and contain FAD as a cofactor [21,34]. CKX proteins are widely detected in various subcellular compartments, such as chloroplast, mitochondria, and so on [35]. Most CKX enzymes are localized in the apoplast, e.g., ZmCKX1 [23], or vacuole, e.g., AtCKX1 and AtCKX3 [36]. Some CKX proteins are shown to be cytosolic enzymes, such as AtCKX7 [37] and ZmCKX10 [23]. AtCKX1 protein is recently reported to be an endoplasmic reticulum (ER) membrane protein [16].

*Bambusa oldhamii*, green bamboo, is a perennial plant in the tropics and subtropics, and bamboo shoot is an economic vegetable in far-eastern Asia [38,39,40,41,42,43,44]. Bamboo is one of the fast-growth timber plants, which are controlled by plant hormones, especially cytokinins [45]. Bamboo cytokinin biosynthesis by the BoAIPT1 isopentenyltransferase enzyme [7] and cytokinin degradation by the BoCKX enzymes may play equally important roles for fine-tuning cytokinin levels in the rapid-dividing tissues. In this study, we cloned and reported three completely sequenced *CKX* genes, including all exon-intron conformations and partial upstream promoter regions. Sequence analysis of the BoCKX1–3 predicts multiple asparagine residues as putative target sites of glycosylation. In addition, several *cis*-acting elements were discovered from the promoters regions of three *BoCKX* genes.

## 2. Materials and Methods

### 2.1. Plant Material

Edible fresh green bamboo shoot mainly harvested between April and September from Mucha mountain areas, Taipei City, Taiwan. Samples were divided into inedible shell and edible shoot and were frozen and stored at −80 °C freezer.

### 2.2. Reagents

DNA ladders and iProof DNA polymerase were obtained from Bio-Rad, Hercules, CA, USA. PrimeStar DNA polymerase mixture was purchased from Takara, Kusatsu, Shiga, Japan. T4 DNA ligase and restriction endonucleases were purchased from New England Biolabs, Ipswich, MA, USA. SeaKem^®^ LE Agarose was obtained from Lonza, Basel, Switzerland. Gel extraction/PCR cleanup kit was purchased from Biotools, New Taipei City, Taiwan. Plasmid mini-prep kit was obtained from Geneaid, New Taipei City, Taiwan. Oligonucleotides synthesis and DNA sequencing services were provided by Tri-I Biotech, New Taipei City, Taiwan.

### 2.3. Total RNA Extraction, cDNA Synthesis, and DIG Labeled Probe Preparation

TRIZOL reagents (Invitrogen, Waltham, MA, USA) were used to extract total RNA from bamboo etiolated shoots and utilized as materials for complementary DNA (cDNA) synthesis using MMLV reverse transcriptase (Invitrogen, Waltham, MA, USA), and used as PCR template. Degenerate primers CKX-F (5′-GGGAGATGGTGACGTGCTCCAA-3′) and CKX-R (5′-CAGCGACACSRMGTAGAACAC-3′) were designed according to the conserved regions of the *OsCKX1* and *OsCKX2* genes from *Oryza sativa* [4]. PCR reaction was carried out at 94 °C for 30 s, 60 °C for 30 s, and 72 °C for 1 min, and then repeated for 30 cycle-reaction. The 900 bp *BoCKX* fragment (Figure S1) was confirmed by DNA sequencing (Tri-I Biotech, New Taipei City, Taiwan). DIG-labeled probe was synthesized by PCR reaction using DIG DNA labeling kit supplied by Roche, Basel, Switzerland.

### 2.4. Genomic Library Screening, Phage DNA Preparation, and DNA Sequencing by Chromosome Walking

A genomic library of green bamboo was previously constructed by Lambda FIX^®^ II/*Xho*I Partial Fill-In Vector Kit (Strategene, San Diego, CA, USA) [7]. Nine to twenty-three kb bamboo genomic DNA fragments were conjugated with a 41.9 kb Lambda FIX^®^ II vector, and the overall phage DNA constructed were between 50 and 65 kb [7]. The DIG-labeled *BoCKX* probe was used to screen the bamboo genomic DNA library. Plaques of phages were transferred onto a Hybond-H^+^ hybridization membrane (MilliporeSigma, Burlington, MA, USA). The processes of hybridization and detection were based on the instructions of the manufacturer (Roche, Basel, Switzerland). Phage DNA was purified from *E. coli* XL1-Blue MRA (P2) by traditional method [46] and digested by *Not*I restriction endonuclease. Phage DNA isolated from positive clones (Lambda midi kit, Qiagene, Hilden, Germany) were further confirmed by DNA sequencing (Tri-I Biotech, New Taipei City, Taiwan), and chromosome walking method was used to obtain complete genomic sequences of the three *BoCKX* genes.

### 2.5. Bioinformatics and Promoter Analysis

Protein sequence alignment was analyzed by Vector NTI Suite 10 Sequence Software (Invitrogen, Waltham, MA, USA). Phosphorylation and glycosylation sites were predicted by NetNGly (http://www.cbs.dtu.dk/servies/NetNGly/, accessed on 15 March 2020) [47] and NetPhos (http://www.cbs.dtu.dk/services/NetPhos/, accessed on 15 March 2020) [48], respectively. The putative *cis*-acting elements and the transcriptional start site were predicted by PlantPAN (retrieved from: http://PlantPAN.itps.ncku.edu.tw, accessed on 25 May 2020) [49] and PlantProm (retrieved from: http://mendel.cs.rhul.ac.uk, accessed on 25 May 2020) [50].

## 3. Results

### 3.1. Cloning of Three BoCKXs Genes by Screening a Bamboo Genomic DNA Library

**Figure 1:**
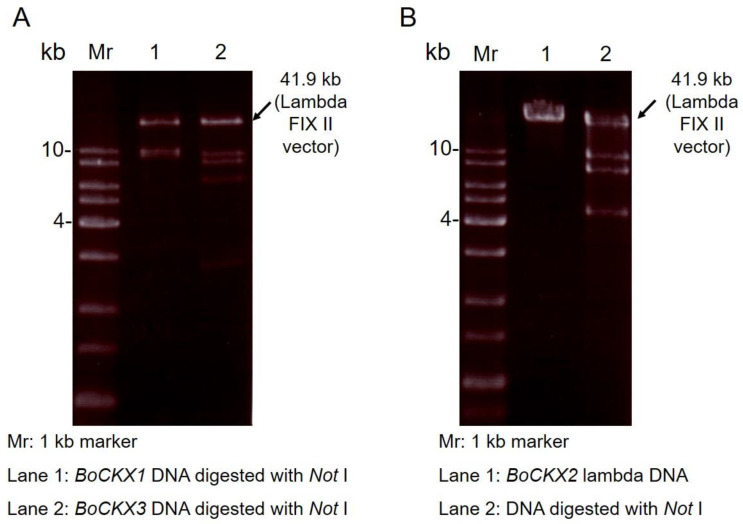
Three *CKX* genes were isolated by screening a bamboo genomic DNA Library. *BoCKX1* (**A**, lane 1), *BoCKX2* (**B**, lanes 1 and 2), and *BoCKX3* (**A**, lane 2) phagemids were purified and then digested by *Not*I. The size of the Lambda FIX II vector (41.9 kb) was indicated.

A Lambda FIX^®^ II genomic library constructed genomic DNA was hybridized and screened by a DIG labeled *BoCKX* probe (Figure S1) [38]. Sequence alignment results showed a high similarity of 64% between the deduced amino acid sequence of the BoCKX probe and ZmCKX1 proteins (Figure S1). Phage DNAs were isolated from positive clones and digested by *Not*I restriction enzyme (Figure 1), followed by DNA sequencing. Three different digestion patterns from four positive clones were observed, indicating that three different *BoCKX* genes were identified. Chromosome walking methodology was performed to obtain the total sequence information of these positive clones. Luckily, three full-length *BoCKX* genes with partial promoter regions were obtained, namely *BoCKX1* (Figure 1A, lane 1), *BoCKX2* (Figure 1B, lanes 1 and 2), and *BoCKX3* (Figure 1A, lane 2). Gene names, *BoCKX1–3*, were designated in the order of DNA sequencing accomplished. These genomic DNA sequences had been deposited at GenBank (Bethesda, MD, USA) with accession numbers, GU263785, GU263786, and GU263787, respectively.

### 3.2. Genomic Organization of the Three BoCKXs Genes

**Figure 2:**
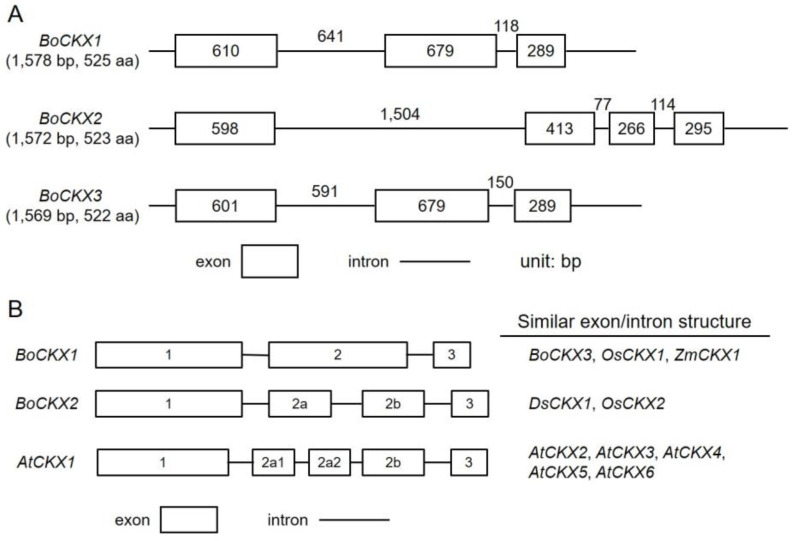
Exon-intron organization of Bamboo *CKX* genes. Introns (lines) and exons (rectangles) were graphed to scale, along with the number of base pairs (bp). Sequences compared (**A**) were isolated from *Bambusa oldhamii* (*BoCKX1*, GU263785; *BoCKX2*, GU263786; and *BoCKX3*, GU263787). Exon-intron structures of the *CKX* genes were compared among different species (**B**).

Phage DNA containing bamboo *CKX* genes were completely sequenced by chromosome walking method, and exon-intron structures of the three *BoCKX* genes were plotted in Figure 2A. The intron-exon organizations of various *CKX* genes in plants were compared and shown in Figure 2B. *BoCKX1* contained a 1578 bp open-reading frame (ORF) and encoded a 525 amino acid polypeptide or a 57.0 kDa protein (Figure 2A). *BoCKX2* contained a 1572 bp ORF and encoded a 523 amino acid polypeptide or a 57.4 kDa protein (Figure 2A). *BoCKX3* contained a 1569 bp ORF and encoded a 522 amino acid polypeptide or a 56.6 kDa protein (Figure 2A).

*BoCKX1* and *BoCKX3* were composed of three exons and two introns (Figure 2A,B), as also observed in other CKX genes, such as *OsCKX1* [4] and *ZmCKX1* [13,14]. Unlike *BoCKX1* and *BoCKX3*, the exon 2 of *BoCKX2* was divided into exons 2a and 2b (Figure 2A,B). As a result, *BoCKX2* possessed four exons and three introns, similar to *Dendrobium sonia DsCKX1* [51] and *OsCKX2* (Figure 2B) [4]. *AtCKX1–6* genes all consisted of four introns and five exons (Figure 2B) [15].

### 3.3. Protein Similarity of CKXs Proteins

**Figure 3:**
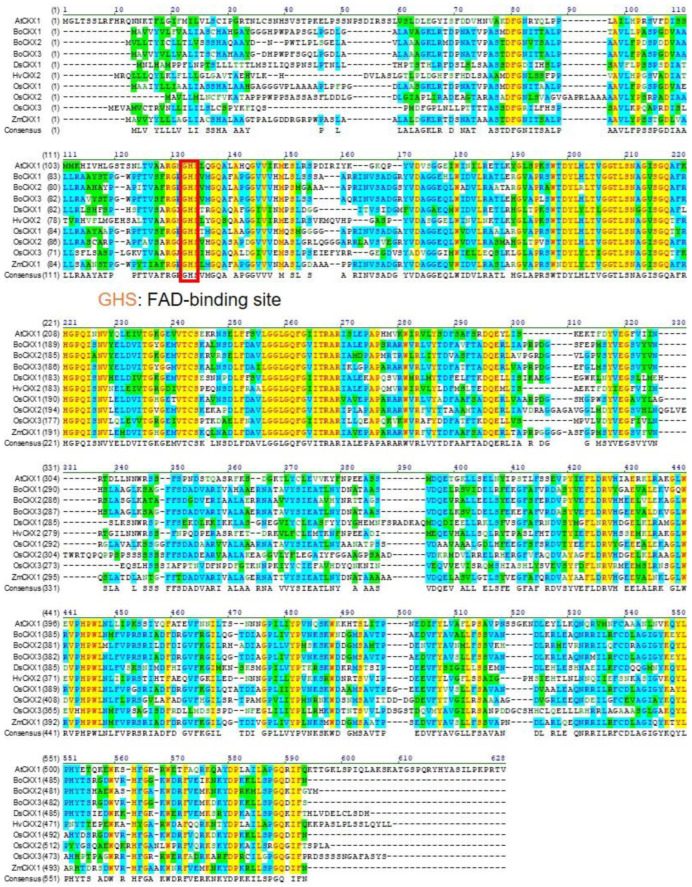
Sequence alignment of the primary structure of BoCKX proteins with CKXs proteins from other species. The sequences shown here were from *Arabidopsis thaliana* (AtCKX1), *Bambusa oldhamii* (BoCKX1–3), *Dendrobium sonia* (DsCKX1), *Hordeum vulgare* (HvCKX2), *Oryza sativa* (OsCKX1–3), and *Zea mays* (ZmCKX1). The conserved FAD-binding site, GHS motif, was indicated.

Protein sequence alignment research revealed that BoCKX proteins had 71–92% identities, with BoCKX1 protein being the most similar to BoCKX3 protein (92% identity; Figure 3 and Table 1).

**Figure 4:**
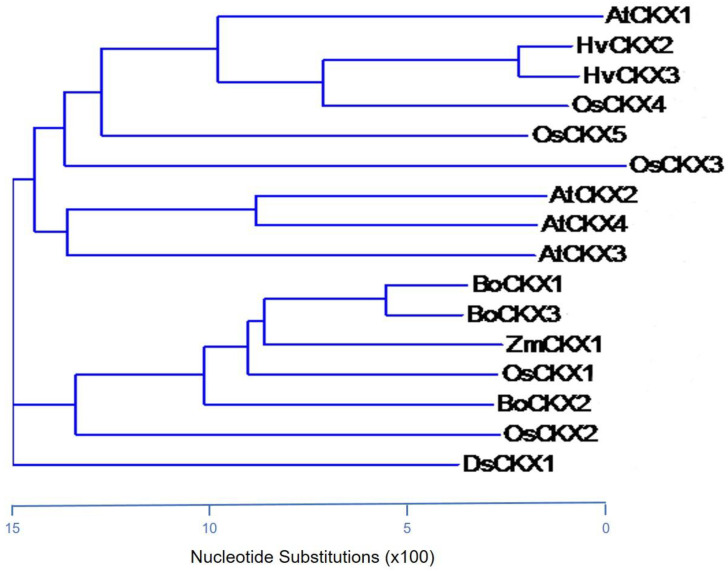
Phylogenetic analysis of CKXs from other plant species by Vector NTI Suite 10 Sequence Software (Invitrogen, Waltham, MA, USA).

The deduced amino acid sequences of CKXs from several plant species underwent phylogenetic analysis (Figure 4). All CKX protein sequences contained a conserved GHS motif as the FAD-binding site and the histidine residue within the GHS motif may be responsible for covalently conjugated with FAD cofactor [34]. OsCKX1 and ZmCKX1 proteins were the closest homolog to BoCKX1 (78% identity) and BoCKX3 (76% identity) proteins, and these genes contained similar exon-intron organization (Figure 2B, Figure 4). Although *BoCKX2* and *OsCKX2* shared identical genomic organization (Figure 2B, Figure 4), the amino acid similarity was only 52% identity.

### 3.4. Analysis of the cis-Acting Elements in BoCKX1

**Figure 5:**
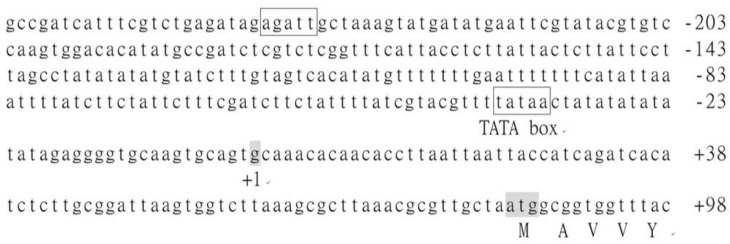
Promoter sequence analysis of the *BoCKX1* gene. The start codon of translation (ATG) was shaded. The predicted transcriptional start site was indicated as “+1”. The putative functional TATA box and one NGATT element were indicated.

To better understand the potential transcriptional regulations, the upstream sequence of the *BoCKX1* was completely sequenced and limited promoter region (−262) was obtained (Figure 5). PlantProm [50] predicted the probable transcriptional start site (+1) of *BoCKX1*, which was situated 83 base pairs from the translational start codon (ATG, Figure 5). Promoter sequence analysis was obtained from PlantPAN [49], and a putative TATA box and an NGATT motif [52] were addressed at −27 and −235 positions, respectively (Figure 5).

### 3.5. Analysis of the cis-Acting Elements in BoCKX2

**Figure 6:**
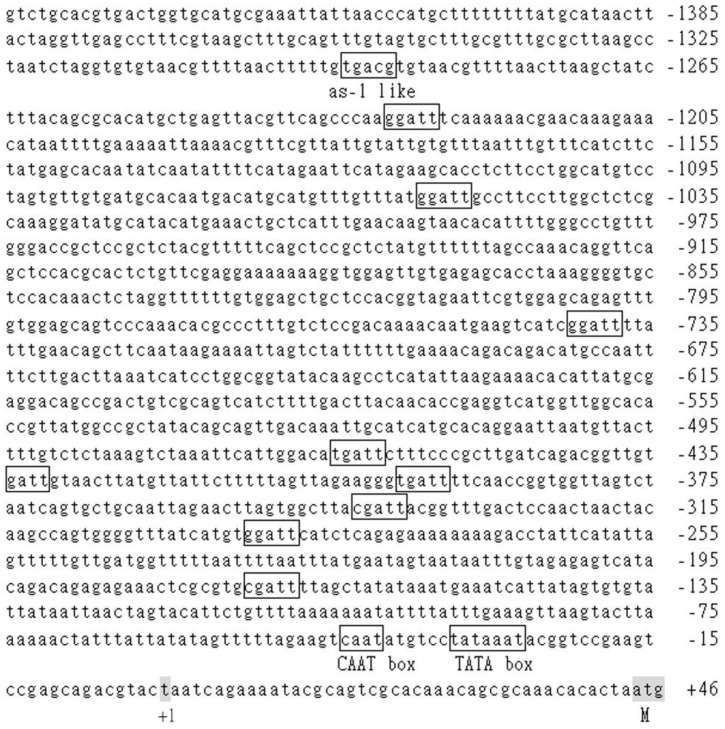
Promoter sequence analysis of the *BoCKX2* gene. The start codon of translation was shaded. The predicted transcriptional start site was indicated as “+1”. The putative functional TATA box, CAAT box, *as-1* like motif and ten NGATT elements were designated.

To better understand the potential transcriptional regulations, the upstream sequence of the *BoCKX2* was completely sequenced and over 1.4 kb promoter region was obtained (Figure 6). PlantProm [50] predicted the probable transcriptional start site (+1) of *BoCKX2*, which was situated 44 base pairs from the translational start codon (ATG, Figure 6). Promoter sequence analysis was performed by PlantPAN [49], and a putative TATA box and CAAT box were addressed at −27 and −40 positions, respectively (Figure 6). Among the promoter region, nine NGATT motifs [52] and one *as-1* like conserved site (−1289) [53] were identified (Figure 6).

### 3.6. Analysis of the cis-Acting Elements in BoCKX3

**Figure 7:**
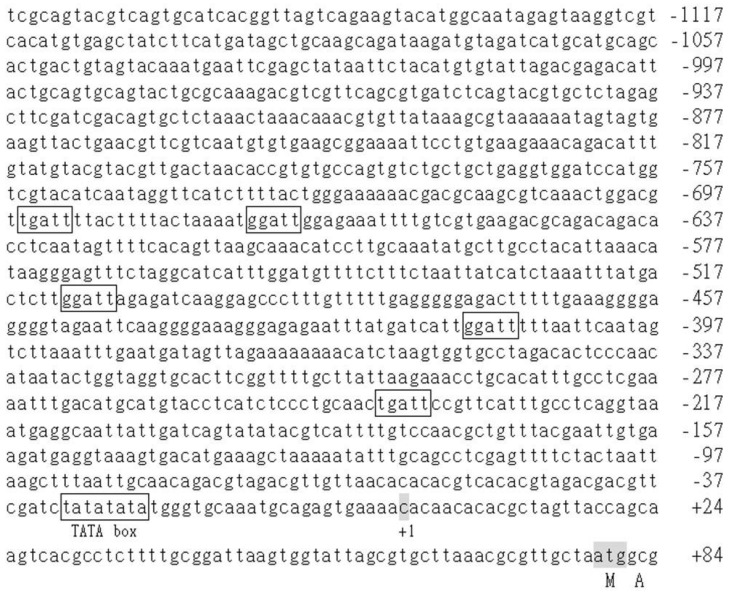
Promoter sequence analysis of the *BoCKX3* gene. The start codon of translation was shaded. The predicted transcriptional start site was indicated as “+1”. The putative functional TATA box and five NGATT elements were designated.

To better understand the potential transcriptional regulations, the upstream sequence of the *BoCKX3* was completely sequenced and ~1.2 kb promoter region was obtained (Figure 7). PlantProm [50] predicted the probable transcriptional start site (+1) of *BoCKX2*, which was situated 79 base pairs from the translational start codon (ATG, Figure 7). Promoter sequence analysis was obtained by PlantPAN [49], a putative TATA box was addressed at −24 position, and five NGATT conserved regions [52] were boxed (Figure 7).

### 3.7. Comparison of Features in Plant CKX Proteins

CKX proteins had been reported to localize at different compartments [16,23,36,37], and some of them were secreted proteins [23]. All three BoCKX proteins were predicted to carry putative signal peptide sequences typical of secretion pathway (Table 2). Cytosolic and vacuolar forms of CKX were identified in other species [16,36], suggesting that there still may be some unknown *BoCKX* genes in *B. oldhamii.*

CKX functions are regulated by post-translational modifications, e.g., glycosylation. All CKX proteins were predicted to be glycoproteins (Table 2), and maize CKXs had been shown to be glycoproteins [14]. By using the NetNGly algorithms, BoCKX1, BoCKX2, and BoCKX3 had seven, five, and six putative glycosylation sites and at least five of them were more than 50% probability (Table 2), implying that BoCKX1–3 may be glycoproteins. In addition to glycosylation, many phosphorylation sites were predicted in BoCKX1–3 (Table 3); however, substantial results are needed.

## 4. Discussion

A multi-gene family of CKX in plants has been reported previously [17,36]. In this study, we identified three cytokinin oxidase/dehydrogenase genes from green bamboo by screening a bamboo genomic DNA library (Figure 1). In the genomic DNA library construction, 9–23 kb bamboo genomic fragments were ligated with a phage vector [7]. Fortunately, four positive clones isolated contained three full coding regions as well as different sizes promoter regions (Figure 1). *BoCKX1* and *BoCKX3* genes had identical exon-intron structures (Figure 2A) as well as 92% amino acid sequence identity (Table 1). The exon-intron structure of the *BoCKX2* was slightly different in comparison with the above two genes (Figure 2A). All three *BoCKX* genes showed similar exon-intron organizations between other *CKX* genes (Figure 2B). In theory, cytoplasmic, ER-membrane, or vascular-localized CKX should exist in green bamboo, and more screening of bamboo genomic DNA can be performed.

Several putative *cis*-acting elements were predicted from promoter regions of the *BoCKX1–3* genes (Figure 5, Figure 6, and Figure 7), and the functions of the motifs were listed in Table 3. All three *BoCKX* genes contained a conserved TATA box and a typical CAAT box was identified in the upstream site of TATA box of the *BoCKX2* [50]. *CKX* gene expression is induced by abiotic stress and plant hormones, such as abscisic acid and cytokinins [25]. As-1 box [53,56,57] and NGATT motif [52,58,59] were present in the 5′-flanking regions of *BoCKX* genes, suggesting that the *BoCKX* gene expressions may be affected by different plant hormones and cytokinin response regulator proteins.

## Figures and Tables

**Table 1:** Amino acids identities (%) among the coding regions of the BoCKX1, BoCKX2, and BoCKX3 proteins.

	BoCKX1	BoCKX2	BoCKX3
BoCKX1	100	78	92
BoCKX2		100	79
BoCKX3			100

**Table 2:** Features of different CKX proteins from various plants.

Gene Name ^a^	No. of Exons	Length (aa)	Mass (kDa) ^b^	Subcellular Localization (PSORT) ^c^	Glycosylation Sites ^d^	Phosphorylation Sites ^e^ Ser Thr Tyr			Reference
*BoCKX1*	3	525	57.0	S ^f^	7/6	11/8	4/1	6/3	This study
*BoCKX2*	4	523	57.4	S	5/5	14/8	5/3	8/3	This study
*BoCKX3*	3	522	56.6	S	6/5	11/8	3/1	8/4	This study
*DsCKX1*	4	536	60.4	S	2/2	^h^ -	-	-	[51]
*OsCKX1*	3	558	59.1	S	2/1	-	-	-	[35]
*OsCKX2*	4	532	56.0	M ^g^	6/5	-	-	-	[35]
*OsCKX3*	5	525	58.0	S	3/3	-	-	-	[35]
*ZmCKX1*	3	534	57.2	S	8/5	-	-	-	[22]

^a^ Bo, Bambusa oldhamii; Ds, Dendrobium sonia; Os, Oryza sativa; Zm, Zea mays. ^b^ Molecular mass calculated with peptide-mass tool (http://ca.expasy.org/tool/peptide-mass.html accessed on 15 March 2020) [54]. ^c^ Subcellular localization predicted with PSORT (http://psort.nibb.ac.jp/ accessed on 15 March 2020) [55]. ^d^ All predicted N-glycosylation sites/predicted glycosylation sites with 50% probability were analyzed by NetNGly (http://www.cbs.dtu.dk/servies/NetNGly/ accessed on 15 March 2020) [47]. ^e^ Phosphorylation site with 50% probability/predicted phosphorylation site with 90% probability was analyzed by NetPhos (http://www.cbs.dtu.dk/services/NetPhos/ accessed on 15 March 2020) [48]. ^f^ S: secretory pathway. ^g^ M: mitochondria. ^h^ -: non-determined.

**Table 3:** Predicted *cis*-acting elements of the *BoCKX* genes and its functions.

*Cis*-Element	Consensus Sequence	Function	References
TATA box	TATAAT	Core promoter *cis*-element of genes in eukaryotes	[50]
CAAT box	CAAT	The CAAT box is a conserved consensus sequence as the binding site of the RNA transcriptional factor	[50]
As-1 box	TGACG	*Activation sequence 1* (*as-1*) is a salicylic acid (SA)- and auxin-responsive element	[53,56,57]
NGATT	N=G/A/C/T GATT	*Arabidopsis* cytokinin response regulators ARR1 binding element	[52,58,59]

## Data Availability

Data are contained in the main article.

## Supplementary Materials

The following supporting information can be downloaded at: https://www.mdpi.com/article/10.3390/cimb45030123/s1, Figure S1: Alignment of the deduced amino acid sequences of *BoCKX*-specific probe and ZmCKX1.
