# Supplementary material for: Cloning of Three Cytokinin Oxidase/Dehydrogenase Genes in Bambusa oldhamii
Source: Curr Issues Mol Biol. 2023 Feb 27;45(3):1902–13. doi: 10.3390/cimb45030123 (PMC10047441; doi:10.3390/cimb45030123)
Supplement: Supplementary file 1 [file cimb-45-00123-s001.zip › cimb-2230820-supplementary.pdf]

Supplementary materials

Cloning of three cytokinin oxidase/dehydrogenase genes in *Bambusa oldhamii*

Chun-Yen Hsieh[1], GiGi Chin Wen Lim[2], and Lu-Sheng Hsieh[2*]

[1]Department of Pathology and Laboratory Medicine, Shin Kong Wu Ho-Su Memorial Hospital, No. 95, Wen Chang Road, Shih Lin District, Taipei City 111, Taiwan.

[2]Department of Food Science, Tunghai University, No. 1727, Sec. 4, Taiwan Boulevard, Xitun District, Taichung 40704, Taiwan.

*Corresponding author E-mail: lshsieh@thu.edu.tw

Tel.: 886-4-23590121#37331

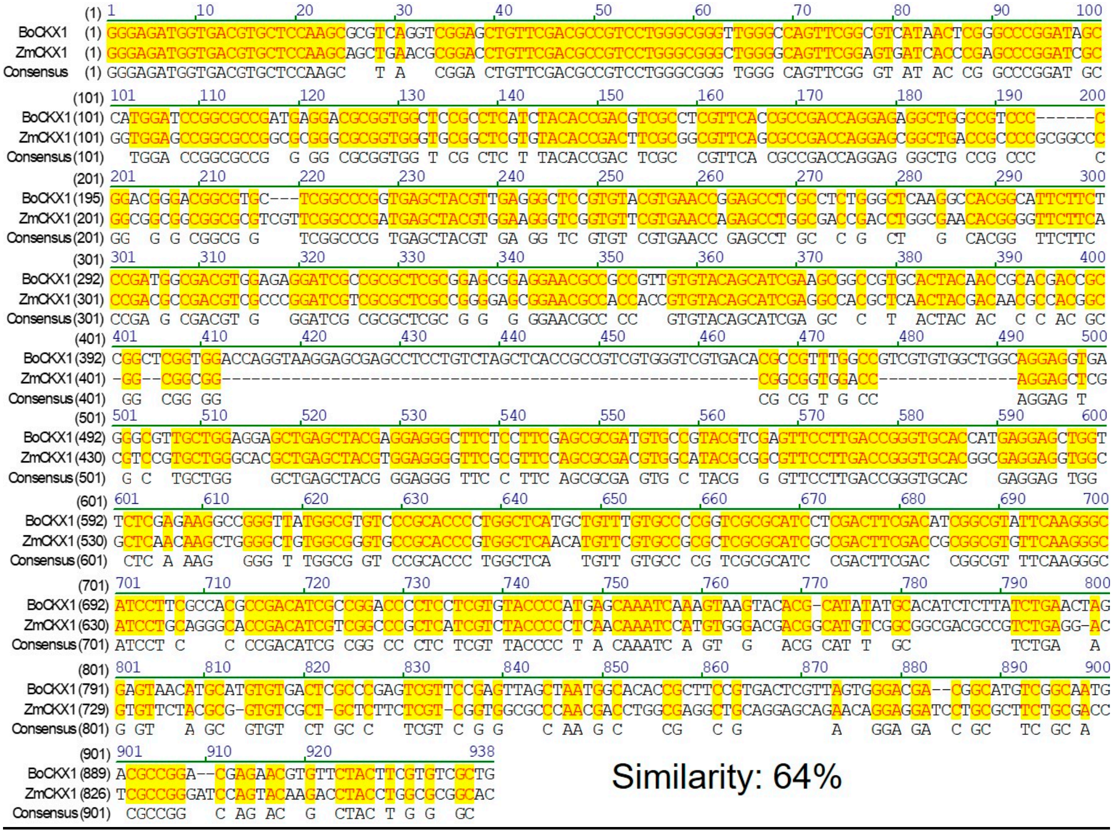

Figure S1. Alignment of the deduced amino acid sequences of *BoCKX*-specific probe and ZmCKX1.
